# Comparison of 3D Conformal Proton Therapy, Intensity-Modulated Proton Therapy, and Intensity-Modulated Photon Therapy for Retroperitoneal Sarcoma

**DOI:** 10.1155/2022/5540615

**Published:** 2022-03-19

**Authors:** Christine Chung, Alexei Trofimov, Judith Adams, Jong Kung, David G. Kirsch, Sam Yoon, Karen Doppke, Thomas Bortfeld, Thomas F. Delaney

**Affiliations:** ^1^John Muir Health, Department of Radiation Oncology, 400 Taylor Boulevard Suite 101, Pleasant Hill, CA 94523, USA; ^2^Massachusetts General Hospital, Mass General Department of Radiation Oncology, 30 Fruit Street, Boston, MA 02114, USA; ^3^Duke Cancer Center, Kirsch Lab, Duke University Medical Center DUMC, Box 91006, Durham, NC 27708, USA; ^4^Columbia University, Department of Surgery, 177 Fort Washington Avenue, Milstein Hospital Building, Room 7-002, New York, NY 10032, USA

## Abstract

**Background:**

External beam radiation therapy (RT) for retroperitoneal sarcoma often requires treatment of large target volumes close to critical normal tissues. Radiation may be limited by adjacent organs at risk (OAR). Intensity-modulated radiation therapy has been shown to improve target coverage and reduce doses to OAR.

**Objectives:**

To compare target coverage and dose to OAR with 3D conformal proton therapy (3D CPT), intensity-modulated proton therapy (IMPT), and intensity-modulated photon therapy (IMXT).

**Methods:**

We performed a comparative study of treatment plans with 3D CPT, IMPT, and IMXT for ten patients with retroperitoneal sarcomas. RT was delivered to 50.4 Gy to the clinical target volume (CTV), the structures considered at risk for microscopic disease.

**Results:**

CTVs ranged from 74 to 357 cc (mean 188 cc). Dose conformity was improved with IMPT, while 3D CPT provided better dose homogeneity. Mean dose to the liver, small bowel, and stomach was reduced with IMPT compared with 3D CPT or IMXT.

**Conclusions:**

IMPT, 3D CPT, and IMXT provide excellent target coverage for retroperitoneal sarcomas. OAR dose is lower with IMPT and 3D CPT, and IMPT achieves the closest conformity. These techniques offer the opportunity for further dose escalation to areas with positive margins.

## 1. Introduction

Retroperitoneal sarcomas are rare tumors, comprising 15% of soft tissue sarcomas [1]. There are approximately 1600 retroperitoneal sarcomas diagnosed each year. The 5-year survival rate is 50–60%, and locoregional recurrence is the most common site of failure [2–4]. Complete resection is the only potentially curative treatment. Patients often present with vague symptoms, and the tumors are frequently large and infiltrative at the time of diagnosis, hindering complete surgical resection. Local recurrence with surgery alone ranges 40–82% at 5 years [5–8], and the posterior extent of the retroperitoneal sarcoma is frequently the site of positive margins or local recurrence [9].

Multimodality therapy has been demonstrated to improve relapse-free survival in sarcoma of the extremities. The utility of radiation in retroperitoneal sarcoma patients has not been demonstrated in a randomized phase III trials, but several series have shown improvements in local control with radiation therapy [10–12]. Preoperative radiation may improve the resectability of the tumor and decrease the risk of intraoperative tumor seeding by sterilization of tumor cells. In addition, the tumor serves to displace bowel preoperatively, so radiation before surgery may decrease the amount of normal tissue irradiated. However, retroperitoneal sarcomas are often large at diagnosis and located adjacent to the small bowel, liver, and kidneys. The proximity to these critical structures limits the preoperative radiation dose to 45–50 Gy due to normal tissue tolerances [12].

Previous studies examined the utility of preoperative external beam radiation for retroperitoneal sarcomas. A phase I study by Pisters et al. examined the use of preoperative radiation therapy to 50.4 Gy with low-dose infusional doxorubicin. The preoperative chemoradiation was well-tolerated and shown to be feasible in combination with surgery and intraoperative electron beam radiation [13]. A study by Mak et al. assessed acute GI toxicity in 56 patients with retroperitoneal sarcoma treated with preoperative radiation therapy and surgery. Three patients (5%) developed grade ≥3 acute GI toxicity, although the dose constraints for the peritoneal cavity were often exceeded [14].

Additional investigators studied the addition of a boost dose to the posterior abdominal wall. Bossi et al. utilized preoperative intensity-modulated radiation therapy to the posterior abdominal wall for retroperitoneal sarcomas. Patients were treated to 50 Gy in 25 fractions to the region considered to be at elevated risk for local relapse. The treatment was well-tolerated without compromising the surgical resectability [15]. Tzeng et al. treated patients with retroperitoneal sarcoma with preoperative radiation therapy to 45 Gy to the whole tumor and a boost of 57.5 Gy to the margin considered at highest risk, with a 2 year local control rate of 80% [16].

Radiation therapy techniques have developed to allow more conformal radiation delivery. Intensity-modulated radiation therapy (IMXT) may improve tumor targeting and sparing of organs at risk, in comparison with standard 3D conformal photon radiation [17]. Proton radiation may also decrease dose to adjacent organs through the deposition of energy at a specified depth, known as the Bragg peak. The use of IMXT or proton radiation may allow for more conformal dose distribution and decreased radiation dose to critical adjacent structures, which could permit the delivery of higher dose to the deep margin of intraabdominal sarcomas [18].

We undertook this study to compare the target coverage and dose to organs at risk (OAR) with 3D conformal proton therapy (3D CPT), intensity-modulated proton therapy, and intensity-modulated photon therapy (IMXT) for ten patients with retroperitoneal sarcomas. In addition, we compared the treatment plans with the addition of a posterior margin boost, using IMXT or IMPT.

## 2. Materials and Methods

After obtaining institutional review board approval, we compared treatment study plans for ten patients with retroperitoneal sarcomas treated at Massachusetts General Hospital Department of Radiation Oncology. Six patients were treated preoperatively, and four patients were treated postoperatively. Each patient had a planning CT scan with images at 2.5–5 mm intervals, and the target volumes and OARs were contoured on the CT images. Six to seven field IMXT plans were designed with the KonRad inverse planning system. The 3D conformal proton plans were developed using the CMS XiO planning software, using two to three fields per plan. The IMPT treatment plans were optimized on the KonRad system, using two to three fields per plan. The IMPT plans differed from the 3D conformal proton plans as a pencil beam scanning technique was utilized to paint the dose in a nonuniform fashion for each IMPT field. A boost plan encompassing the posterior margin was developed for patients treated preoperatively, in order to calculate the impact of this additional boost dose on the organs at risk. Boost plans were designed using IMXT and IMPT. The treatment plans were optimized to maximize the coverage of the clinical target volume (CTV), while minimizing dose to the OARs.

**Figure 1:**
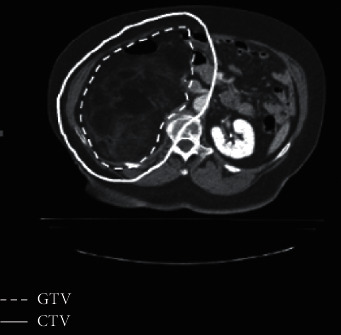
Gross tumor volume and the intended clinical target volume planned standardly in the preoperative setting.

The CTV consisted of the retroperitoneal structures considered at risk for microscopic residual disease in patients undergoing postoperative radiation therapy. Among patients treated preoperatively, CTV was defined as the gross tumor with a 1.5 cm volume expansion. A smaller margin was applied, if there was no significant risk of invasion, due to the presence of a fascial, peritoneal, bony, or organ margins. The PTV consisted of CTV with a 0.5 cm expansion. The boost volume included the tumor abutting the posterior abdominal wall with a 2 cm margin (Figure 1). The GTV, CTV, and PTV were held constant for all treatment plans. The normal tissue contours were similarly held constant.

**Figure 2:**
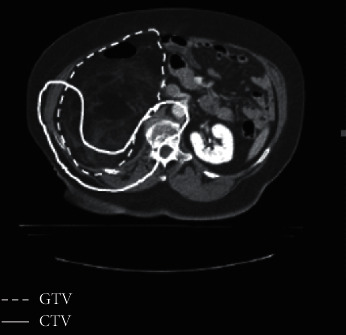
Gross tumor volume and intended clinical target volume planned according to our protocol.

The objective of the treatment plans was to deliver 50.4 Gy in 1.8 Gy per fraction to 100% of CTV and >95% of PTV. For patients treated preoperatively, a boost dose of 9 Gy was given to the posterior margin, for a total planned dose of 59.4 Gy. For patients treated postoperatively, a boost of 16.2 Gy was planned, for a total dose of 66.6 Gy (Figure 2). Proton doses were expressed in GyRBE, equivalent to the proton dose in Gray multiplied by a relative biological effectiveness factor of 1.1 for protons.

The following normal tissue constraints were used: 50% of each kidney <20 Gy, 50% of the liver <30 Gy, and 90% of the small bowel <45 Gy. The maximal dose permitted to the duodenum, stomach, and colon was 50.4 Gy, respectively. The maximal dose to the spinal cord was 54 Gy, and the maximal dose to the cauda equina was 50 Gy.

Treatment comparisons were performed using a variety of parameters. Dose-volume histograms (DVHs) were generated for CTV and OARs of each plan. The plans were assessed with a variety of parameters, including the dose delivered to 95% of the target tissue (*D*_95%_), the homogeneity coefficient, and the conformity index. The homogeneity coefficient (HC) was defined as (*D*_5%_–*D*_95%_)/*D*_95%_ and should equal zero if there is no inhomogeneity within the target. The conformity index (CI) was calculated by V95%/PTV.

The Wilcoxon rank sum test was used for statistical calculations.

## 3. Results

Ten patients with retroperitoneal sarcomas were included in the study. Patient characteristics are given in Table 1.

The CTV and PTV coverage was adequate for all of the treatment plans. The mean PTV was 778 cc of tissue (range 160–2552). The mean CTV was 521 cc (range 74–1927). All plans achieved a dose of >50.4 Gy to 95% of the target volume.

The 3D conformal proton plans demonstrated the highest degree of homogeneity within the target volume, with a mean homogeneity coefficient of 4.0% (range 2.1–7.1%) (Table 2). This was a statistically significant improvement from the mean IMXT homogeneity coefficient of 6.8% (range 5.8–8.6%, *p*=0.015). In comparison, the IMPT homogeneity coefficient of 7.5% (range 6.7–8.8%) did not differ significantly from the IMXT plan (*p*=0.121).

The IMPT plans demonstrated the greatest conformity with the planned target volume, with a conformity index of 1.15 (range 1.05–1.30) (Table 3). This represented a significant improvement over the IMXT plans (mean 1.35, range 1.19–1.50, *p*=0.005). The 3D CPT had a lower degree of conformity in comparison with IMXT plans (mean 1.78, range 1.37–2.34, *p*=0.032).

**Figure 3:**
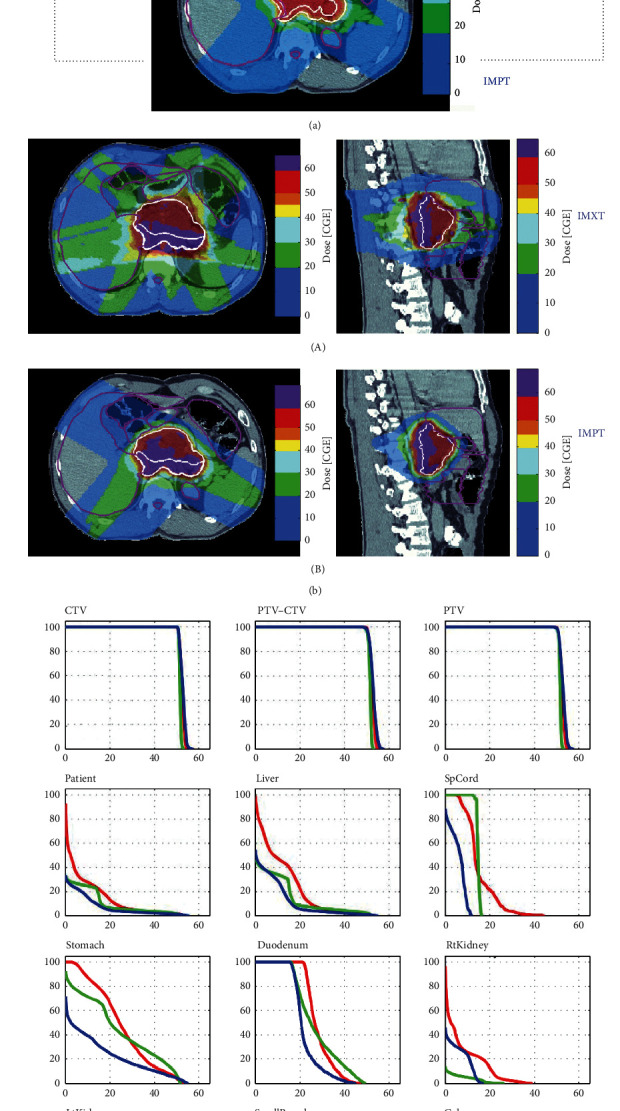
(a) Axial CT slices with isodose lines comparing IMXT, 3D CPT, and IMPT for myxoid liposarcoma patient. (b) Axial and sagittal CT slices with isodose lines comparing IMXT (A) and IMPT boost plan (B) for a myxoid liposarcoma patient. (c) Dose-volume histogram for myxoid liposarcoma patient.

The plans were also assessed for the dose to the organs at risk (Table 4). The mean dose to the liver was 11.8 Gy with IMXT, 6.61 GyRBE with 3D CPT, and 5.73 GyRBE with IMPT. The mean dose to the stomach was 15.4 Gy with IMXT, 11.8 GyRBE with 3D CPT, and 7.85 GyRBE with IMPT. The mean small bowel dose was 11.4 Gy with IMXT, 7.13 GyRBE with 3D CPT, and 3.9 Gy with IMPT.  Figure 3(a) shows axial CT slices with isodose lines of a patient with myxoid liposarcoma, demonstrating improved conformity and decreased radiation dose to the adjacent normal structures, including the liver and small bowel, with the IMPT plan.

Doses to the organs at risk were also calculated for the boost plans. With the preoperative boost, the mean dose to the liver was 16.7 Gy with IMXT and 9.2 GyRBE with IMPT. The stomach dose was 28.4 Gy with IMXT and 16.8 GyRBE with IMPT. The small bowel dose was 13.4 Gy with IMXT and 5.8 GyRBE with IMPT.  Figure 3(b) shows axial CT slices demonstrating decreased dose to organs at risk with the IMPT boost plan. The dose-volume histograms in Figure 3(c) further demonstrate the lower dose to critical adjacent organs with the use of IMPT.

## 4. Discussion

This study represents the largest treatment planning study to date for retroperitoneal sarcomas. IMPT, 3D CPT, and IMXT treatment plans were successfully generated for each of the ten patients. Each of the three treatment modalities achieved excellent target coverage to doses of 50.4 Gy, while maintaining normal tissue constraints. The IMPT and IMXT plans resulted in higher inhomogeneity within the target, but all techniques yielded clinically acceptable outcomes. IMPT achieved the closest degree of conformity to the target volume.

As suggested by previous studies [18], proton radiation decreased the volume of adjacent normal tissue receiving radiation, potentially lowering toxicity to the bowel, stomach, and small bowel. In this study, IMPT and 3D CPT provided the lowest radiation dose to the OAR. In comparison with IMXT, 3D CPT reduced the mean radiation dose to the liver by 5.19 Gy, and IMPT reduced the dose to the liver by 6.07 Gy. Proton radiation also improved mean radiation dose to the stomach and small bowel. In comparison with IMXT, 3D CPT reduced radiation dose to the stomach by 3.6 Gy and IMPT reduced the gastric dose by 7.55 Gy. The mean dose to the small bowel was decreased by 6.27 Gy with the use of 3D CPT and by 7.6 Gy with IMPT.

A prior study by Swanson et al. compared three-dimensional conformal proton radiation, 3D conformal photon radiation, and intensity-modulated radiation plans for eight patients with retroperitoneal and intraabdominal sarcomas [18]. The IMXT and 3D conformal proton plans provided greater conformity and dose homogeneity than 3D conformal photon radiation plans. The 3D conformal proton plans offered better sparing of the small bowel and kidneys, suggesting potential improvement in the therapeutic ratio. Our current study adds to this body of knowledge by including IMPT plans and examining the addition of a boost to the posterior margin.

These dosimetric analyses are limited in predicting true clinical benefit, though can be helpful in guiding the development of prospective trials. Several single institution studies using these advanced radiation techniques have been performed. Preoperative proton radiation and/or intensity modulated radiation therapy were combined with aggressive surgical resection in a study by Yoon et al. Patients with close posterior margins were treated with intraoperative electron radiation therapy. At a median follow-up of 33 months, only 2 patients (10%) with primary disease developed a local recurrence [9].

A phase I trial by Delaney et al. in 2017 examined the use of preoperative IMPT and surgery for eleven patients with retroperitoneal sarcoma [19]. The patients were treated with 50.4 GyRBE, with a simultaneous integrated boost up to 63 GyRBE. The dose escalation was tolerated well, with acute toxicity that was ≤grade 2. A single patient developed late grade 3 hydronephrosis postoperatively. At a median follow-up of 18 months, no patient who underwent resection developed a local recurrence. A phase II study is underway to assess the efficacy and safety of preoperative IMPT for these patients.

The prospective studies of radiation in retroperitoneal sarcoma have been disappointing. The American College of Surgeons Oncology Group opened a randomized trial of preoperative radiation therapy versus surgery alone for retroperitoneal sarcomas, but the trial was closed due to poor accrual [20]. A recent randomized trial by the European Organization for Research and Treatment of Cancer (EORTC) examined the use of 50.4 Gy of preoperative radiation therapy and surgery versus surgery alone [21]. 266 retroperitoneal sarcoma patients were randomized, and 198 patients (74.5%) had liposarcoma. At a median follow-up of 43 months, the median abdominal recurrence-free survival was 4.5 years among patients treated with radiation and surgery and 5 years in the surgery only arm (*p*=0.95). A greater number of patients in the radiation and surgery group suffered grade 3-4 adverse events (24%) vs. patients treated with surgery alone (10%) [22, 23].

Dosimetric analyses, such as this one and the study by Swanson et al. [18], suggest that the use of more conformal radiation methods such as IMXT or proton therapy may improve the therapeutic ratio, allowing for dose escalation to the areas at greatest risk of local recurrence while minimizing toxicity. However, further prospective clinical trials are required to determine if techniques such as IMXT or IMPT will translate into improved outcomes for patients.

Furthermore, the utilization of radiation technology is not the sole factor in treating these complex patients. Consensus guidelines established by a panel of expert radiation oncologists noted that the margin at greatest risk of local recurrence may be difficult to predict. While delivery of a boost dose to the posterior abdominal wall may be technically feasible, additional toxicity may occur if the boost dose is given to the pancreas or duodenum. The panel did not recommend a boost dose as standard practice, but only on a clinical protocol or at experienced centers [24].

The use of more conformal radiation therapies, as well as improved targeting of the margin at highest risk of recurrence, may help to improve local control while reducing toxicity for patients with retroperitoneal sarcoma.

## 5. Conclusion

This study demonstrates that proton therapy provided a benefit to the organs at risk among patients treated preoperatively for retroperitoneal sarcomas. IMPT, 3D CPT, and IMXT treatment plans were able to meet the organ at risk constraints as outlined in the consensus guidelines. Selective dose escalation to the retroperitoneal margins of these tumors was also possible using IMXT and IMPT techniques. Further data from ongoing clinical trials will provide further information regarding the utility of a posterior margin boost and the role of proton therapy in the treatment of retroperitoneal sarcoma.

## Figures and Tables

**Table 1:** Patient characteristics.

Age	Sex	Histology	Planning target volume size (cc)
69	Female	Sarcoma	410
62	Male	Sarcoma	324
59	Male	Liposarcoma	160
45	Female	Sarcoma	194
39	Female	Chondrosarcoma	712
33	Male	Myxoid liposarcoma	287
71	Female	Dedifferentiated liposarcoma	2552
51	Female	Connective tissue sarcoma	1502
51	Male	Dedifferentiated liposarcoma	1142
46	Female	Connective tissue sarcoma	598

**Table 2:** PTV inhomogeneity coefficient_._

	Range (%)	Mean (%)	*P* value (Wilcoxon rank sum test)
IMXT	5.8–8.6	6.8	
3D CPT	2.1–7.1	4.0	0.015
IMPT	6.7–8.8	7.5	0.121

Inhomogeneity coefficient = radiation dose delivered to 5% of the target tissue (*D*_5%_)−dose to 95% of the target tissue (*D*_95%_)/*D*_95%_.

**Table 3:** PTV conformity index.

	Range	Mean	*P* value (Wilcoxon rank sum test)
IMXT	1.19–1.50	1.35	
3D CPT	1.37–2.34	1.78	0.032
IMPT	1.05–1.30	1.15	0.005

PTV conformity index (CI) = target tissue receiving 95% of the dose/planning target volume (V95%/PTV).

**Table 4:** Dose to organs at risk.

	Dmean to the liver (*n* = 8)	Preop boost (*n* = 3)
IMXT	0.94–24.6 Gy, mean 11.8 Gy	12.0–24.6 Gy, mean 16.7 Gy
3D CPT	1.1–20.9 Gy,1.2 mean 6.61 Gy (*p*=0.01)	—
IMPT	0.99–18.6 Gy, mean 5.73 Gy (*p*=0.03)	2.8–18.6 Gy, mean 9.2 Gy

	Dmean to the stomach (*n* = 8)	Preop boost (*n* = 3)
IMXT	4.03–44.2 Gy, mean 15.4 Gy	13.3–43.6 Gy, mean 28.4 Gy
3D CPT	0–50.0 Gy, mean 11.8 Gy (*p*=NS)	—
IMPT	0–36.5 Gy, mean 7.85 Gy (*p*=0.02)	3.5–35.2 Gy, mean 16.8 Gy

	Dmean to the small bowel (*n* = 8)	Preop boost (*n* = 4)
IMXT	1.71–24.8 Gy, mean 13.4 Gy	4–21.5 Gy, mean 11.4 Gy
3D CPT	2.4–16.0 Gy, mean 7.13 Gy (*p*=0.01)	—
IMPT	0.82–12.3 Gy, mean 5.80 Gy (*p*=0.03)	0.68–7.6 Gy, mean 3.9 Gy

## Data Availability

The data used to support the findings of this study are available from the corresponding author upon request.
